# Causes of death and conditional survival estimates of long-term lung cancer survivors


**DOI:** 10.3389/fimmu.2022.1012247

**Published:** 2022-09-23

**Authors:** Qun Zhang, Yuan Dai, Hongda Liu, Wenkui Sun, Yuming Huang, Zheng Gong, Shanlin Dai, Hui Kong, Weiping Xie

**Affiliations:** ^1^ Department of Respiratory and Critical Care Medicine, The First Affiliated Hospital of Nanjing Medical University, Nanjing, China; ^2^ Department of General Surgery, The First Affiliated Hospital of Nanjing Medical University, Nanjing, China; ^3^ Department of Thoracic Surgery, The First Affiliated Hospital of Nanjing Medical University, Nanjing, China; ^4^ The Jackson Laboratory, Bar Harbor, ME, United States

**Keywords:** lung cancer, conditional survival, death hazard, causes of death, immunotherapy

## Abstract

**Introduction:**

Lung cancer ranks the leading cause of cancer-related death worldwide. This retrospective cohort study was designed to determine time-dependent death hazards of diverse causes and conditional survival of lung cancer.

**Methods:**

We collected 816,436 lung cancer cases during 2000-2015 in the SEER database, after exclusion, 612,100 cases were enrolled for data analyses. Cancer-specific survival, overall survival and dynamic death hazard were assessed in this study. Additionally, based on the FDA approval time of Nivolumab in 2015, we evaluated the effect of immunotherapy on metastatic patients’ survival by comparing cases in 2016-2018 (immunotherapy era, n=7135) and those in 2013-2016 (non-immunotherapy era, n=42061).

**Results:**

Of the 612,100 patients, 285,705 were women, the mean (SD) age was 68.3 (11.0) years old. 252,558 patients were characterized as lung adenocarcinoma, 133,302 cases were lung squamous cell carcinoma, and only 78,700 cases were small cell lung carcinomas. TNM stage was I in 140,518 cases, II in 38,225 cases, III in 159,095 cases, and IV in 274,262 patients. 164,394 cases underwent surgical intervention. The 5-y overall survival and cancer-specific survival were 54.2% and 73.8%, respectively. The 5-y conditional survival rate of cancer-specific survival is improved in a time-dependent pattern, while conditional overall survival tends to be steady after 5-y follow-up. Except from age, hazard disparities of other risk factors (such as stage and surgery) diminished over time according to the conditional survival curves. After 8 years since diagnosis, mortality hazard from other causes became higher than that from lung cancer. This critical time point was earlier in elder patients while was postponed in patients with advanced stages. Moreover, both cancer-specific survival and overall survival of metastatic patients in immunotherapy era were significantly better than those in non-immunotherapy era (P<0.001), indicating that immunotherapeutic intervention indeed bring remarkable benefits to advanced lung cancer patients.

**Conclusions:**

Our findings expand on previous studies by demonstrating that non-lung-cancer related death risk becomes more and more predominant over the course of follow-up, and we establish a personalized web-based calculator to determine this critical time point for long-term survivors. We also confirmed the survival benefit of advanced lung cancer patients in immunotherapy era.

## Introduction

With an estimated number of 236,740 new cases and 130,180 deaths in 2022, lung cancer contributes to the leading cancer-related death in United States (1). Non–small-cell lung cancer (NSCLC) and small-cell lung cancer (SCLC) are two predominant histological subtypes. Although subtype-specific incidence and mortality have been well described (2, 3), limited survival information exists for long-term survivors. With achievements in NSCLC treatment including targeted therapies and immunotherapies, lung cancer survival has been remarkably improved in the past decade (4).

During clinical practice, oncologists are more frequently consulted with the question “how many extra years could I survive” instead of “how many years could I survive”. To answer this question, conditional survival is a better choice than accrued survival. Conditional survival (*t*|*s*) is defined as the probability of surviving further *t* years, given that a patient has already survived *s* years after the diagnosis of a specific disease (5). There have been studies characterized conditional survival of specific lung cancer populations such as unresectable stage III NSCLCs (6) and elder patients with stage I to III SCLC (7) that received with chemo-radiation therapies. Nevertheless, systematical conditional survival assessment targeting the nationwide lung cancer population is lacking.

In the present study, lung cancer patients in Surveillance, Epidemiology, and End Results (SEER) database during 2000-2015 were collected. The primary purpose of our study was to compare the conditional survival of lung cancer cases with different demographic and clinicopathological characteristics. In addition, during data analyses, we found that conditional CSS (cancer-specific survival) was improved time-dependently, while the conditional OS (overall survival) tend to be steady in long-term survivors. Therefore, we further determined the critical point when death hazard from lung cancer became lower than that from other risks. Considering the clinical significance of immunotherapies in lung cancer treatment (8), especially for those who could not undergo surgery, we also investigated whether it brought survival benefits to patients in SEER dataset.

## Methods

### Patients

The SEER database is an annual-updated, population-based cancer data from 19 cancer registries encompassing 35% of the US population as of 2021. For relative long-term follow-up, the 2000-2015 SEER database was retrieved for patients with primary lung cancer (n=816,436). Patients with consistent SEER AYA site record of lung cancer and without other malignancies before lung cancer were included in this study. We excluded patients diagnosed only by death certification or autopsy. Patients with unclear TNM stage or survival month less than 1 were also excluded. According to the criteria above, there were 612,100 cases enrolled in this retrospective cohort study.

To compare the survival difference of metastatic patients in immunotherapy era with those in non-immunotherapy era, we enrolled another cohort from 2013 to 2018. Patients were divided with the median year, 2016, after which Nivolumab was approved by FDA for metastasic lung cancer patients treatment (9). Because of the shorter follow-up of patients in 2016-2018 compared with that in 2013-2015, we only included patients within 12 months survival. Finally 42061 metastatic lung cancer patients in nonimmunotherapy era 2013-2015 and another 7135 metastatic lung cancer patients in immunotherapy era 2016-2018 were collected in this analysis.

### Statistical analyses

Deaths were classified as due to lung cancer or other causes. Smoothed annual hazards of death due to lung cancer, other causes, or any cause were evaluated respectively. Univariate logistic regression analyses and multivariate Cox proportional hazards regression analyses were used to determine prognostic risk factors for overall survival and cancer-specific survival, including age, sex, histology, TNM stage and surgery. Conditional survival analysis was conducted to evaluate time-dynamic survival for patients who have survived specific years (5). For example, conditional survival (5|4) was calculated as CS(5|4)=S(4 + 5)/S (4), indicating an additional 5-year survival possibility of patients who have survived 4 years since lung cancer diagnosis. Kaplan-Meier method was employed to calculate survival rates for diverse subgroups. SPSS Version 23.0 and R Software were used for statistical analysis. Two-sided P value <.05 was considered statistically significant.

### Ethics

Institutional Review Board approval of this study was obtained from The Ethic Committee of Nanjing Medical University First Affiliated Hospital.

## Results

### Baseline characteristics

**Figure 1:**
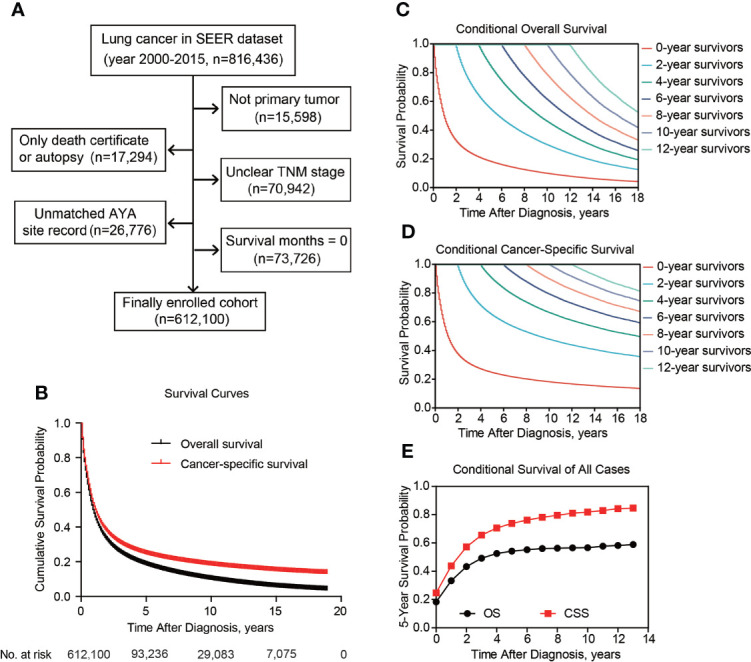
Patient inclusion and survival of lung cancer patients. Patients inclusion and exclusion flow **(A)**. Overall and cancer-specific survival of all included cases **(B)**. Overall **(C)** and cancer-specific **(D)** conditional survival of lung cancer patients with Kaplan-Meier analysis. **(E)**, The 5-year overall and cancer-specific conditional survival rate of enrolled patients.

After patients’ exclusion, 612,100 patients diagnosed with lung cancer during 2000-2015 were queried from SEER database (**Figure 1A**). As shown in **Table 1**, 146,122 patients (23.9%) aged ≤ 60 yrs, 294,539 patients (48.1%) aged 61-75 yrs, and the other 171,439 patients (28.0%) were elder than 75 yrs at the time of diagnosis. There were more male cases (n=326,395, 53.3%) than females (n=285,705, 46.7%). As for the histological type, 252,558 (41.3%) cases were lung adenocarcinoma (LUAD), 133,302 (21.8%) cases were lung squamous cell carcinoma (LSCC), 78,700 (12.9%) cases were small-cell lung cancer (SCLC), and 147,540 cases (24.1%) were classified as other non-small-cell lung cancers (NSCLC) including large cell carcinoma, adenosquamous carcinoma, etc. The percentages of each TNM stages were showed respectively in **Table 1** (stage I for 23.0%, n=140,518; II for 6.2%, n=38,225; III for 26.0%, n=159,095; IV for 44.8%, n=274,262). Although surgical information was unclear for 0.6% patients (n=3,723), 26.9% patients (n=164,394) underwent surgical resection while the other 72.5% patients (n=443,983) did not receive surgical intervention.

**Table 1:** Clinicopathologic characteristics and overall survival probabilities.

Variables	Patients, No. (%)	OS, mean (SD), yr	5-Y OS rate (%)	P value^a^	HR	95% CI	P value^b^
Age							
≤ 60 yrs	146,122 (23.9)	3.92 (0.02)	21.97%		Reference		
61-75 yrs	294,539 (48.1)	3.20 (0.01)	20.01%		1.23	1.22-1.24	<.001*
> 75 yrs	171,439 (28.0)	2.04 (0.01)	12.33%	<.001*	1.65	1.64-1.66	<.001*
Sex							
Female	326,395 (53.3)	2.59 (0.01)	15.03%		Reference		
Male	285,705 (46.7)	3.60 (0.01)	22.10%	<.001*	0.82	0.82-0.83	<.001*
Histology							
LUAD	252,558 (41.3)	3.93 (0.01)	24.64%		Reference		
LSCC	133,302 (21.8)	2.95 (0.01)	18.45%		1.20	1.19-1.21	<.001*
Other NSCLC	147,540 (24.1)	2.48 (0.01)	13.75%		1.22	1.21-1.23	<.001*
SCLC	78,700 (12.9)	1.54 (0.01)	6.49%	<.001*	1.18	1.17-1.19	<.001*
TNM stage							
Stage I	140,518 (23.0)	6.87 (0.02)	47.89%		Reference		
Stage II	38,225 (6.2)	4.95 (0.03)	32.37%		1.37	1.35-1.38	<.001*
Stage III	159,095 (26.0)	2.58 (0.01)	13.97%		1.77	1.75-1.78	<.001*
Stage IV	274,262 (44.8)	1.11 (0.01)	3.66%	<.001*	3.13	3.10-3.16	<.001*
Surgery							
Yes	164,394 (26.9)	7.12 (0.02)	49.66%		Reference		
No	443,983 (72.5)	1.54 (0.01)	6.68%		2.21	2.20-2.23	<.001*
Unknown	3,723 (0.6)	2.14 (0.06)	11.02%	<.001*	2.01	1.98-2.11	<.001*

CI, confidence interval; HR, hazard ratio; LSCC, lung squamous cell carcinoma; LUAD, lung adenocarcinoma; NSCLC, non–small-cell lung cancer; OS, overall survival; SCLC, small-cell lung cancer; SD, standard deviation; * indicates P<0.05.

a Data significance was compared by log-rank test.

b Data significance was compared by Cox-regression test.

### Survival and conditional survival

The 5-y OS of entire cohort was 54.2%, and the 5-y CSS was 73.8% (**Figure 1B**). According to Kaplan-Meier survival analysis, univariate analysis and multivariate Cox proportional hazards regression analysis, all the retrieved characteristics exhibited statistical significance (**Tables 1**, **2**). In detail, patients aged 61-75 yrs (HR, 1.23; 95% CI, 1.22-1.24) or aged elder than 75 yrs (HR, 1.65; 95% CI, 1.64-1.66) were exhibited with worse OS compared with younger patients. Compared with LUAD, LSCC (HR, 1.20; 95% CI, 1.19-1.21), other NSCLC (HR, 1.22; 95% CI, 1.21-1.23), and SCLC (HR, 1.18; 95% CI, 1.17-1.19) all showed worse OS. Compared with TNM stage I patients, the HR (95% CI) of stage II, stage III, and stage IV were 1.37 (1.35-1.38), 1.77 (1.75-1.78), and 3.13 (3.10-3.16), respectively. As expected, patients without surgical treatment showed worse OS (HR, 2.21; 95% CI, 2.20-2.23). Consistent with previous studies, female patients seemed to have better prognosis (HR, 0.82; 95% CI, 0.82-0.83). As for CSS, all the above variables showed consistent tendencies and significances with OS (**Table 2**).

**Table 2:** Cancer-specific survival probabilities.

Variables	Patients, No. (%)	CSS, mean (SD), yr	5-Y CSS rate (%)	P value^a^	HR	95% CI	P value^b^
Age							
≤ 60 yrs	146,122 (23.9)	4.84 (0.02)	25.69%		Reference		
61-75 yrs	294,539 (48.1)	4.66 (0.02)	26.37%		1.15	1.14-1.16	<.001*
> 75 yrs	171,439 (28.0)	3.67 (0.02)	20.52%	<.001*	1.44	1.43-1.45	<.001*
Sex							
Male	326,395 (53.3)	3.92 (0.01)	21.21%		Reference		
Female	285,705 (46.7)	5.10 (0.02)	28.48%	<.001*	0.84	0.83-0.84	<.001*
Histology							
LUAD	252,558 (41.3)	5.51 (0.02)	31.30%		Reference		
LSCC	133,302 (21.8)	4.70 (0.02)	26.73%		1.17	1.16-1.18	<.001*
Other NSCLC	147,540 (24.1)	3.75 (0.02)	19.66%		1.21	1.21-1.22	<.001*
SCLC	78,700 (12.9)	2.0 (0.02)	8.72%	<.001*	1.20	1.18-1.21	<.001*
TNM stage							
Stage I	140,518 (23.0)	10.49 (0.03)	62.74%		Reference		
Stage II	38,225 (6.2)	7.15 (0.05)	42.18%		1.67	1.65-1.70	<.001*
Stage III	159,095 (26.0)	3.57 (0.02)	18.82%		2.34	2.32-2.37	<.001*
Stage IV	274,262 (44.8)	1.40 (0.01)	5.13%	<.001*	4.27	4.22-4.31	<.001*
Surgery							
Yes	164,394 (26.9)	10.11 (0.02)	60.67%		Reference		
No	443,983 (72.5)	2.16 (0.01)	10.15%		2.34	2.32-2.36	<.001*
Unknown	3,723 (0.6)	3.22 (0.11)	16.79%	<.001*	2.17	2.09-2.26	<.001*

CI, confidence interval; CSS, cancer-specific survival; HR, hazard ratio; LSCC, lung squamous cell carcinoma; LUAD, lung adenocarcinoma; NSCLC, non–small-cell lung cancer; OS, overall survival; SCLC, small-cell lung cancer; SD, standard deviation; * indicates P<0.05.

a Data significance was compared by log-rank test.

b Data significance was compared by Cox-regression test.

**Figure 2:**
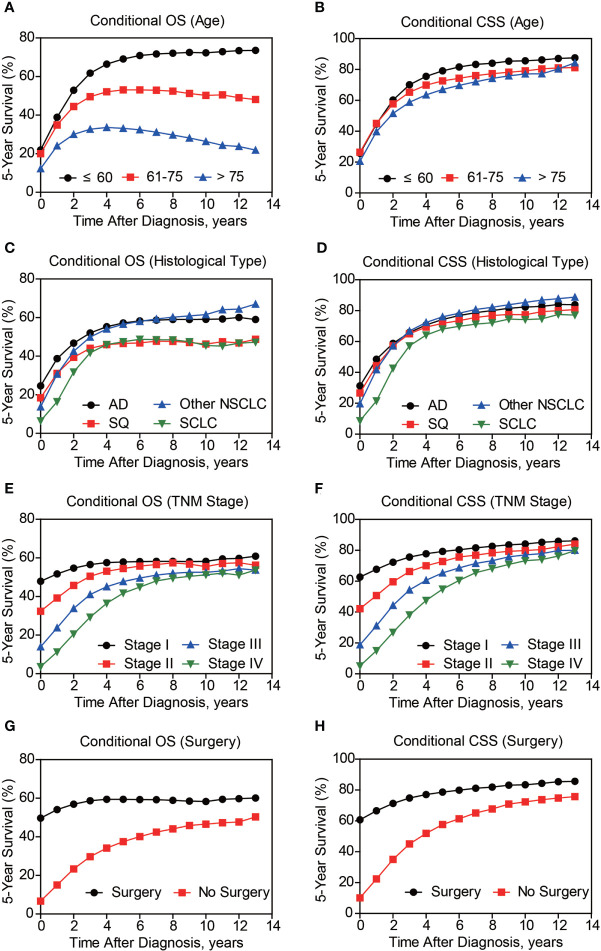
Subgroup analyses of 5-year conditional survival of lung cancer patients. The 5-year conditional overall survival (OS) and conditional cancer-specific survival (CSS) analyses based on patients’ age **(A, B)**, histological type **(C, D)**, TNM stage **(E, F)**, and surgical treatment **(G, H)**.

Besides cumulative survival, conditional OS (**Figure 1C**) and conditional CSS (**Figure 1D**) were evaluated in our study. Compared with 5-y conditional survival curves, conditional survival rate of CSS was increased in a time-dependent manner, while conditional OS tended to be steady as approximately 50% after 5-y follow-up (**Figure 1E**). Stratification analyses were next performed to figure out factors that might affect conditional survival (**Figure 2**). Considering age was perhaps the most nonnegligible factor affecting conditional survival time, it was reasonable that conditional OS of elder lung cancer patients exhibited as an inverted U-shaped curve, which peaked at conditional (5|4). This could also explain why age was the only factor that resulted in drifting apart curves (**Figure 2A**). Nevertheless, conditional CSS of patients with different ages were similar and increased time-dependently (**Figure 2B**).

**Figure 3:**
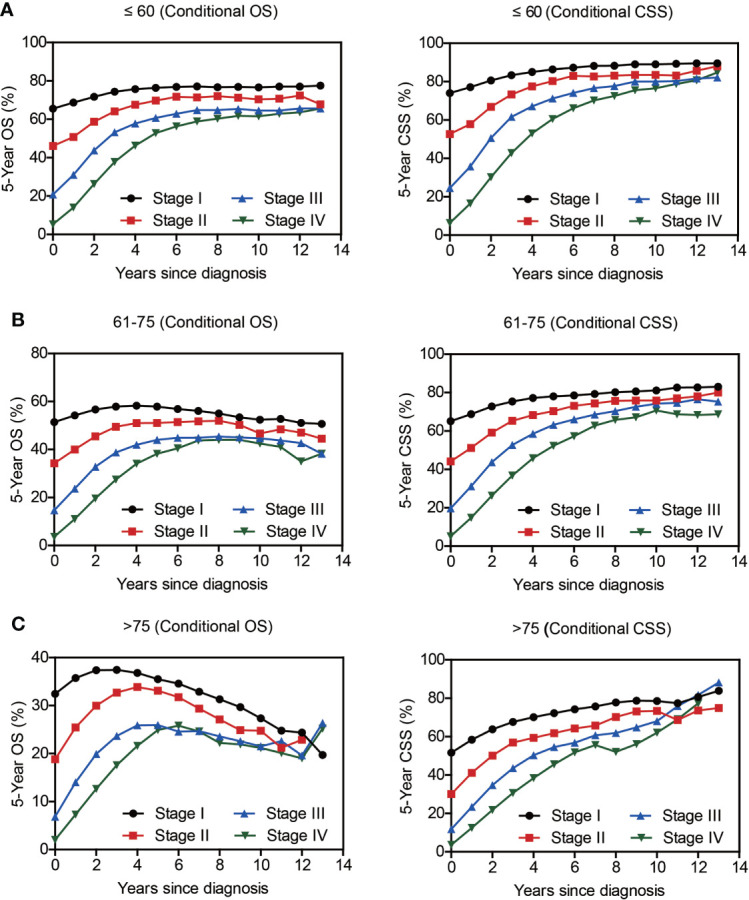
The 5-year conditional survival rate of lung cancer as per the TNM Stage and Age. Subgroup analyses of 5-year conditional survival rate based on patients’ TNM stage of those aging ≤ 60 yrs **(A)**, 61-75 yrs **(B)**, or > 75 yrs **(C)**.

Compared with NSCLC, although SCLC patients exhibited poorer accrued OS, its conditional OS was equivalent to that of LSCC patients since 3-year postdiagnosis, while was still lower than that of LUAD and other NSCLC types (**Figure 2C**). As for conditional CSS, the disparity between SCLC and NSCLC was becoming smaller in a time-dependent manner (**Figure 2D**). Similarly, conditional survival disparities between patients in different TNM stages evaporated over time (**Figures 2E, F**), indicating that stage may be no longer a risk factor for long-term survivors. On the other hand, we also compared the effects of stage on conditional survival rate of cases within the same age range (**Figure 3**), which obtained a consistent conclusion. The discrepancies of conditional OS and CSS in different TNM stages vanished over time for all patients of various age periods (**Figures 3A-C)**.

**Figure 4:**
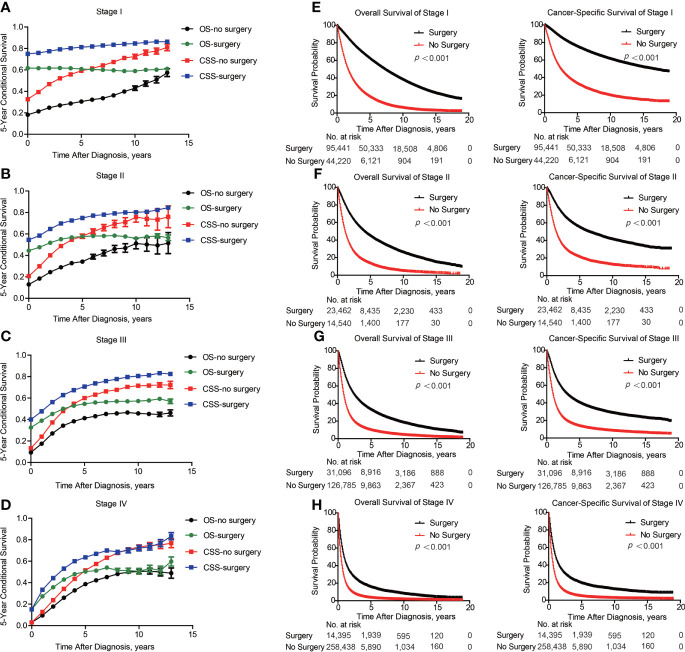
Subgroup analyses of survival based on surgical treatment in lung cancer patients with Different stages. Subgroup analyses of 5-year conditional survival rate based on surgical treatment or not of patients in stage I **(A)**, stage II **(B)**, stage III **(C)** and stage IV **(D)**. Subgroup analyses of overall and cancer-specific survival with Kaplan-Meier analysis based on surgical treatment or not of patients in stage I **(E)**, stage II **(F)**, stage III **(G)** and stage IV **(H)**.

**Figure 5:**
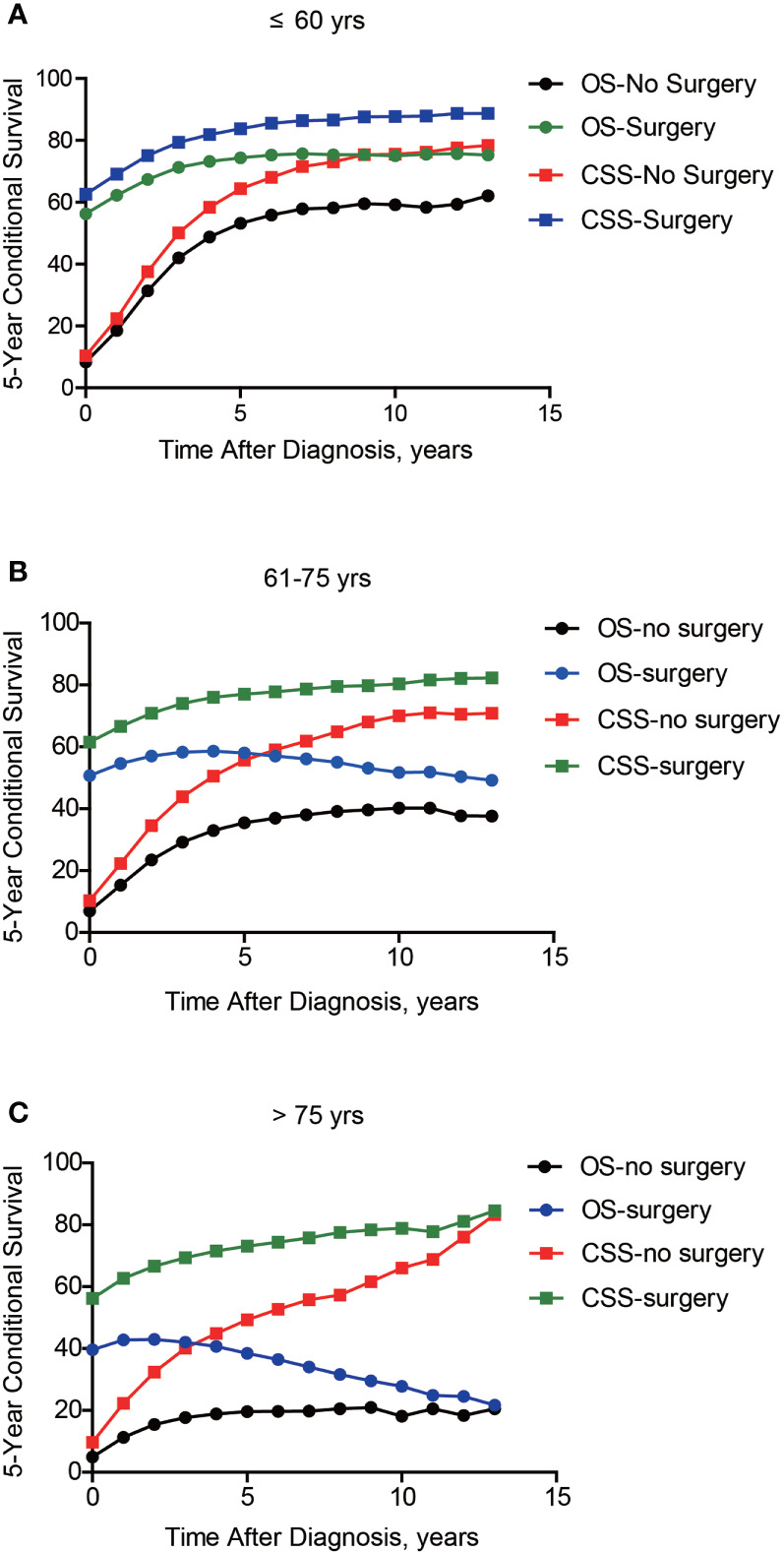
Subgroup analyses of 5-year conditional survival based on surgical treatment in lung cancer patients with different ages. Subgroup analyses of 5-year conditional survival rate based on surgical treatment or not of those aging ≤ 60 yrs **(A)**, 61-75 yrs **(B)**, or > 75 yrs **(C)**.

Of note, even conditional survival curves were becoming more and more approximating in patients with or without surgical treatment (**Figures 2G, H**), the differences were still remarkable even at conditional (5|13). For better understanding, we further conducted subgroup analyses of surgical treatment. Interestingly, except patients in stage III, conditional survival disparities between patients with or without surgical treatment evaporated over time in subgroups of stage I, II and IV (**Figures 4A-D**). Additionally, significant differences of OS and CSS between surgery and no surgery patients were revealed in each stage subgroup (**Figures 4E-H**). This finding was also applicable when comparing the effect of surgery in patients within different age-subgroups, especially in patients over 75 years old (**Figures 5A-C**). Taken together, surgical resection was strongly recommended for patients when surgery is possible, which remarkably affect prognosis even for advanced stages.

### Time-dependent alteration of death risk

**Figure 6:**
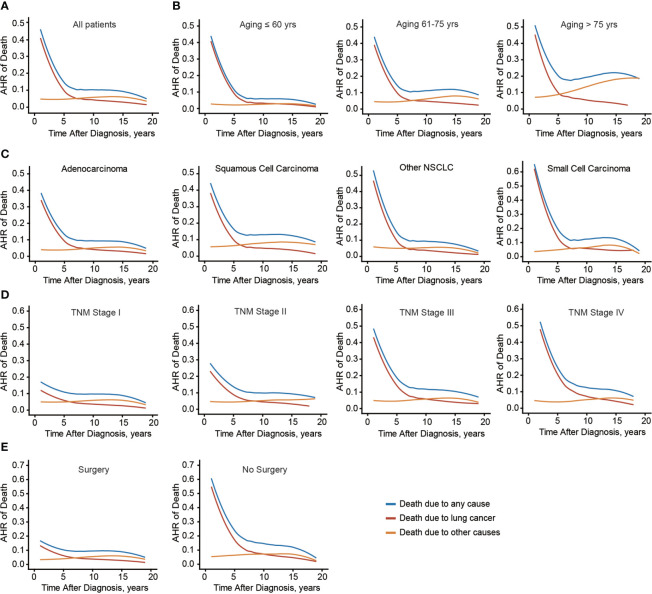
Annual hazard rate of death due to any cause, lung cancer, and other causes. Annual hazard rate (AHR) of death due to different causes of the entire cohort **(A)**, or based on patients’ age **(B)**, histological type **(C)**, TNM stage **(D)**, and surgical treatment **(E)**.

Considering the distinct effects of age on conditional OS and CSS, the time-dynamic death hazard from lung cancer or other causes was analyzed (**Figure 6A**). Consistent with conditional survival data, death risk from lung cancer decreased dramatically in the first 5 years (from 46.0% to 8.5%). Meanwhile, after 8 years, death hazard contributed by other causes became higher than that by lung cancer. The above findings engaged us to further investigate whether these time points were different in subgroups. As a result, the critical points were 13-year, 8-year, and 5-year in patients aged ≤ 60, 61-75, and > 75 yrs, respectively (**Figure 6B**). Interestingly, the time point of LSCC was the earliest one (6-year) among all lung cancer histological subtypes (**Figure 6C**), emphasizing the histological difference. Moreover, this critical point was delayed in patients with more advanced TNM stages (**Figure 6D**). Initial death hazard in patients without surgical treatment was significantly higher than that in cases received surgery (**Figure 6E**). Additionally, the critical point was postponed in those who did not receive surgery. Taken together, the death hazard from non-lung cancer causes in patients with elder age, LSCC subtype, early tumor stage, or received surgical intervention exceeded that from lung cancer at an earlier follow-up date.

To provide better clinical instruction on follow-up focus, we combined all the confounding factors and established a web-based calculator targeting this critical time point (https://lccs.shinyapps.io/lungcancercauses/). For example, for male LSCC patients aged ≤ 60, with poor differentiation grade (grade III), T2N0M0, and received surgical treatment, the critical time point was 10-year (**eFigure 1**), indicating that for those specific patients, subsequent visit should be more focus on other death risk factors since then.

**Figure 7:**
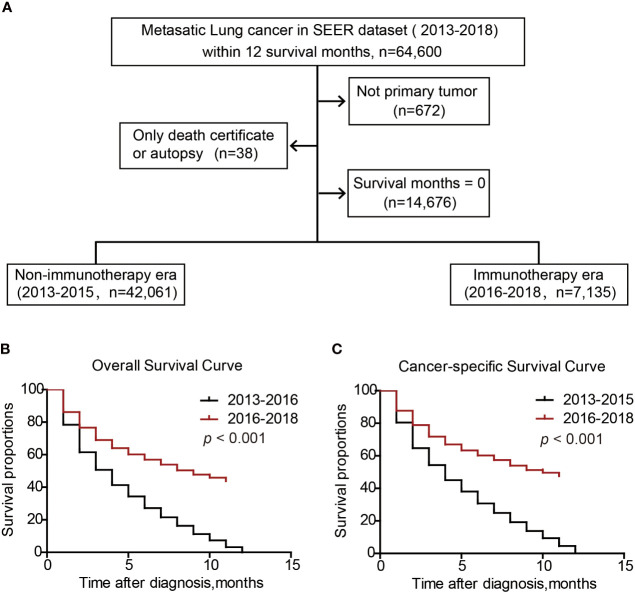
Survival benefits of metastatic lung cancer patients in immunotherapeutic era. Patients inclusion and exclusion flow **(A)**. Overall survival **(B)** and cancer-specific survival **(C)** of metastatic lung cancer patients.

To compare the survival difference of metastatic patients in immunotherapy era with those in non-immunotherapy era, we enrolled another cohort from 2013 to 2018 (**Figure 7A**). Among them, patients within 2016-2018 were defined as immunotherapy era (n=7135), while those within 2013-2016 were defined as non-immunotherapy era (n=42061). Overall survival analysis indicated that the median OS time of metastatic lung patients in non-immunotherapy era was 4 months, while increased to 9 months in immunotherapy era (**Figure 7B**, P<0.001). Consistently, the median CSS time of metastatic lung patients in non-immunotherapy era was 4 months, while increased to 10 months in immunotherapy era (**Figure 7C**, P<0.001). Taken together, the immunotherapy bring significant survival benefits to metastatic lung cancer patients.

## Discussion

Lung cancer ranks the leading cause of cancer-related death worldwide (10, 11). Currently, predominant focus is paid on short-term lung cancer survivors due to its highly malignant characteristics (12). However, due to the advance of novel diagnostic and therapeutic methods, the prognosis of lung cancer patients is improving as indicated by surveys targeting different populations. A previous study showed that 5-year survival of lung cancer in Japan population between 1970s and 2000s increased from 6.0% to 30.9% in females and from 7.2% to 19.6% in males (13). Similarly, 5-year survival of lung cancer in USA increased from 10.7% in 1973 to 19.8% in 2010 (2). A Hungarian study also measured a 5.3% increase in 5-year lung cancer survival from the period 2011–2012 to 2015–2016 (14). Additionally, an increased lung cancer incidence is associated with air pollution, especially in developing countries (15). Therefore, there will be increasing patients suffer from lung cancer in the near future.

As a more dynamic and personalized survival evaluation method compared with cumulative survival, conditional survival is now valued in clinical consulting for long-term survivors. Studies had observed that lung cancer cases with worse prognostic characteristics at the time of diagnosis exhibited better improvements on conditional survival over time (16). In other words, the unfavorable effects of certain risk factors, such as advanced tumor stage, may gradually decline in patient population over the course of follow-up. However, many reports only focused on specific lung cancer patients such as those who were surgical treated (17, 18) or those who received prophylactic cranial irradiation (19). Therefore, conditional survival data seems distinct perhaps due to particular and limited cases.

Here we initially estimated the nationwide population-based conditional survival of lung cancer in USA using SEER dataset, which could help physicians and patients better predict the following survival time. In this study, time debilitated the roles of all risk factors on affecting conditional cancer-specific survival of lung cancer patients. This would be an encouragement to patients who possessed unfavorable clinicopathological characteristics on that they might show comparable survival benefits after several years. Consistently, annual death hazard from lung cancer decreased from 46.0% to 8.5% within the first 5 years since diagnosis. Similar observations were reported in pancreatic ductal adenocarcinoma (PDAC) patients on that the PDAC-related mortality dramatically decreased within 6 years and then remained constant (20).

One of the most important concerns of cancer survivors is their death hazard from the malignancy. On one hand, although cancer-specific mortality exceeds that of other diseases in the first few years, this cancer-related death hazard is diminishing in a time-dependent pattern. On the other hand, effects of other survival determinants accumulate in different periods of life course, resulting in an increasing important risk of other causes such as heart disease in lung cancer patients over the course of follow-up. In that case, our doctors are highly attached importance to the medical comorbidities and specific health assessments for long-term survivors. Considering that the requirement for follow-up focuses and clinical instructions for long-term survivors are distinct from short-term survivors (21), determining examination balance is indispensable for long-term survivors.

In the current study, we aim to evaluate conditional survival of lung cancers and to ascertain if there is a critical time point when the death hazard from other causes exceeds that from lung cancer. Accordingly, we demonstrate that this critical point is distinct in patients with different clinical characteristics. Firstly, the younger at the time of diagnosis, the later the critical point reaches. This is reasonable because death hazard from other causes, such as cardiovascular diseases, increases with ageing. Indeed, a previous study reported that the probability of dying of lung cancer is equal to that of cardiovascular diseases after 7-year survival in stage I NSCLC who accepted lobectomy aged 70-79 years, while this time point shortens to 5-year in cases aged 80 years or older (22).

Secondly, intrinsic cancer phenotypes are also influential. For example, patients with LSCC reach this critical point at 6-year, while other histological types are later. Besides, the number of years before reaching this critical point is positively correlated with TNM stage. This is due to the fact that the initial death risk from lung cancer is significantly smaller in earlier-stage cases than that in advanced-stage cases. Thirdly, our findings emphasize the significant benefit of surgical treatment on controlling lung-cancer related death hazard. For those who didn’t receive surgical intervention, lung cancer-related death remains the leading hazard until 10-year after diagnosis. Therefore, we strongly recommend that surgical resection should be performed at the utmost effort.

Currently, there are now several web-based calculators to estimate different prognostic aspects of lung cancers such as accrued survival (23), conditional survival (24), and conditional site-specific recurrence (25). Nonetheless, no public calculator is available on determining individual follow-up critical point for long-term lung cancer survivors. Therefore, we establish a web-based calculator to identify personalized critical point for lung cancer patients of various age, sex, histology, differentiation grade, TNM stage and surgical treatment, which can help provide statistical evidence of death hazard during clinical practice.

Increasing evidence promoted the application of immunotherapies, such as PD-1/PD-L1 inhibitors, in advanced lung cancer treatment (26). Here we briefly divided metastatic lung cancer patients from SEER dataset into non-immunotherapeutic era (2015 and before) and immunotherapeutic era (after 2015) based on the FDA approval time of Nivolumab in 2015 (9, 27). As expected, our results confirmed that metastatic lung cancer patients in immunotherapeutic era showed a 5-month longer OS time and 6-month longer CSS time than those in non-immunotherapeutic era.

## Limitation

According to the data issued by American Lung Association (www.lung.org), there is a large variance regarding lung cancer survivals in different states. For example, survival rate ranks highest in Connecticut as 28.8%, while Alabama ranked lowest as 18.4%. Meanwhile, African Americans with NSCLC were reported to have worse conditional survival compared with other ethnic groups, indicating that conditional survival vary by ethnicity (28, 29). These regional and racial variations deserve further investigations.

## Conclusions

Conditional cancer-specific survival is continuously improved in long-term lung cancer survivors, especially for those accepted immunotherapeutic treatment. Lung cancer ceased to be the major cause of death for patients who have survived 8 years since diagnosis. For elder lung cancer patients or early staged patients, more attention should be paid on other death risks from an earlier time point.

## Data availability statement

The raw data supporting the conclusions of this article will be made available by the authors, without undue reservation.

## Ethics statement

The studies involving human participants were reviewed and approved by The Ethic Committee of Nanjing Medical University First Affiliated Hospital. Written informed consent to participate in this study was provided by the participants’ legal guardian/next of kin.

## Author contributions

QZ: Data curation, Writing preparation. YD: Analysis, Methodology. HL and WS: Investigation, Resources, Software. YH: Supervision. ZG: Validation. SD: Conceptualization. HK: Project administration. WX: Project administration. All authors contributed to the article and approved the submitted version.

## Funding

This study was supported by National Natural Science Foundation of China (82002438), Fellowship of China Postdoctoral Science Foundation (2020M671396), Fellowship of Jiangsu Postdoctoral Science Foundation (2020Z239).

## Conflict of interest

The authors declare that the research was conducted in the absence of any commercial or financial relationships that could be construed as a potential conflict of interest.

## Publisher’s note

All claims expressed in this article are solely those of the authors and do not necessarily represent those of their affiliated organizations, or those of the publisher, the editors and the reviewers. Any product that may be evaluated in this article, or claim that may be made by its manufacturer, is not guaranteed or endorsed by the publisher.
