# Supplementary material for: Causes of death and conditional survival estimates of long-term lung cancer survivors:
Source: Front Immunol. 2022 Sep 23;13:1012247. doi: 10.3389/fimmu.2022.1012247 (PMC9537558; doi:10.3389/fimmu.2022.1012247)
Supplement: Supplementary file 1 [file DataSheet_1.docx]

**Causes of Death and Conditional Survival Estimates of Long-term Lung Cancer Survivors**


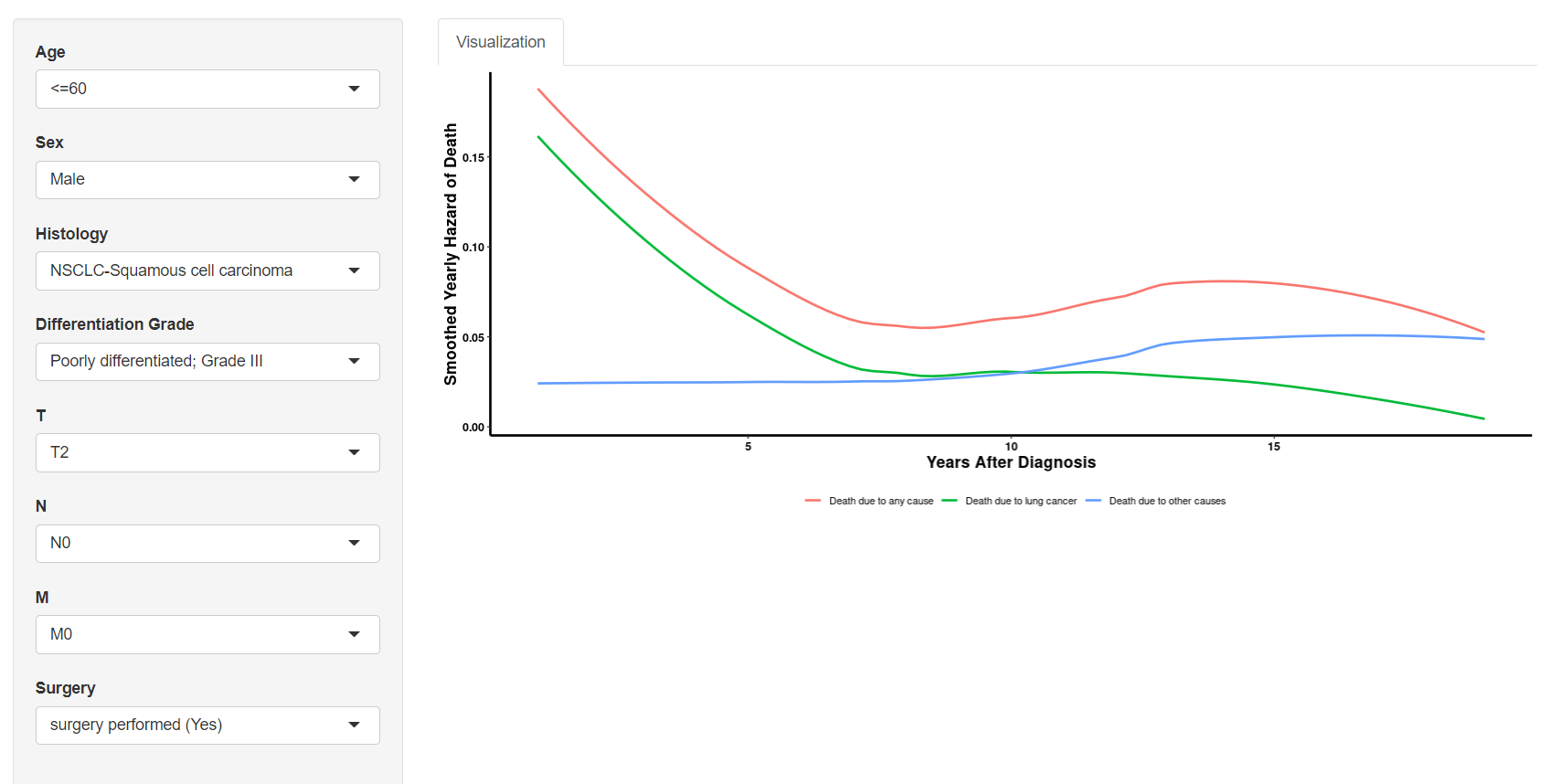


**eFigure 1. Representative Screenshot of the Web-Based Calculator to Determine Critical Time Point.**

This Figure is generated from website <https://lccs.shinyapps.io/lungcancercauses/> to help establish when the death risk from other causes will exceed that from lung cancer for different lung cancer patients. It indicates that, for male LSCC patients aged ≤ 60, with poor differentiation grade (grade III), T2N0M0, and received surgical treatment, the critical time point was 10-year, subsequent visit should more focus on other death risks since then.
